# Applicability of next-generation risk assessment generic PBK models and their performance within the chemical domain

**DOI:** 10.1007/s00204-026-04443-7

**Published:** 2026-05-25

**Authors:** M. Spaenig, M. W. Ashraf, M. Bouhifd, C. Drake, T. Sobanski, S. E. Escher

**Affiliations:** 1 Fraunhofer Institute for Toxicology and Experimental Medicine, Hannover, Germany; 2 University of Veterinary Medicine, Hannover, Germany; 3 European Chemical Agency (ECHA), Helsinki, Finland

**Keywords:** PBK, ADME, Applicability domain, Regulatory use, Uncertainty, Chemicals

## Abstract

Physiologically based kinetic (PBK) models are a cornerstone of in vitro to in vivo extrapolation and are therefore central to next-generation risk assessment (NGRA). This study assesses the performance of a generic, high-throughput PBK model for 26 organic chemicals spanning a wide range of physicochemical properties. Model predictions based on standard in silico and in vitro input parameters were benchmarked against published, chemical-specific PBK models and in vivo kinetic data. Predicted plasma maximum concentrations (Cmax) were within tenfold of in vivo values for 50% of the chemicals and within threefold for 31%. Published PBK models generally showed closer agreement with in vivo Cmax, likely because they incorporate in vivo-derived kinetic parameters or chemical-specific kinetic processes. An analysis of the chemical space revealed a lipophilicity-dependent bias, where Cmax for lipophilic compounds tended to be underpredicted, whereas hydrophilic compounds were overpredicted. No other consistent trends were observed with respect to physicochemical or ADME descriptors, possibly reflecting the limited dataset size. Overall, while the generic PBK model workflow appears suitable for NGRA applications, the observed lipophilicity-dependent bias indicates that chemical-specific corrections or refinements may be required for more accurate predictions.

**Supplementary Information:**

The online version contains supplementary material available at 10.1007/s00204-026-04443-7.

## Introduction

Physiologically based kinetic (PBK) models allow the translation of external doses to time specific data of tissue and blood concentrations in living organisms, using a set of ordinary differential equations and knowledge about species- and substance-specific kinetic parameters. These PBK models, although often sharing the same underlying sources of information, are versatile instruments that can be constructed and utilized in a variety of applications, including in vitro to in vivo extrapolation (IVIVE), kinetic predictions in special populations e.g., infants and pregnant women, and interspecies extrapolation, to name a few (Najjar et al. 2022; Thépaut et al. 2023; Zhang et al. 2023). These models range from relatively generic models, requiring a limited number of input parameters paired with assumptions and simplifications, to more sophisticated models, which often include mechanistic and complex structures that require specialized input parameters.

In a generic model, resembling the presumably most crucial features of a substance’s kinetics, absorption is based on fraction absorbed and first order uptake rate, distribution by partitioning relative to fraction unbound in plasma, hepatic clearance, and excretion by a default kidney clearance (Punt et al. 2022b). A simple generic model keeps compound specific input parameters as well as physiological processes to a minimum to capture the key features of substance kinetics. Therefore, it typically relies on several assumptions e.g., that the intestinal absorption is approximated by the fraction absorbed and a first-order uptake rate; distribution is represented by partitioning relative to the unbound fraction in plasma; hepatic clearance is accounted for; and excretion is modeled using a default renal clearance.

Whether these models resemble substance specific kinetics in an adequate manner requires a validation of individual model workflows. Typical metrics to assess the goodness of fit of these models are the maximum plasma concentration (C_max_), its timepoint after exposure (T_max_) and the overall systemic exposure indicated by the area under the plasma concentration curve (AUC) with a goodness-of-fit often being indicated by predictions being within twofold (Paini et al. 2017).

For next generation risk assessment (NGRA), generic PBK models have the potential to assess bioavailability and kinetics without the use of animals (Moxon et al. 2020; Paini et al. 2019). In vitro to in vivo extrapolation applies in vitro biokinetic as well as PBK based reverse dosimetry to extrapolate human exposure levels from new approach methodologies (NAM) based in vitro test batteries (Breen et al. 2021; Clewell et al. 2008; Wetmore et al. 2015). Regulatory acceptance of PBK models is an ongoing process, currently tackling important questions and hurdles (Paini et al. 2017).

Concordance with the in vivo kinetics data is considered the gold standard of PBK model validation. However, when this data is not available, the predictions of PBK models may often be considered uncertain and may therefore lack trust for regulatory assessments. In such scenarios, the uncertainty in these models can be minimized by the use of a validated modeling workflow for compounds with similar absorption, distribution, metabolism and excretion (ADME) properties (EMA 2018). Also, several guidance documents have been developed to guide the development and regulatory use of PBK models (OECD 2021; WHO 2010).

As required in NGRA, a workflow for PBK models containing in silico predicted and in vitro measured input parameters has been applied in previously published studies and has shown promising results. In a recent study by Geci et al. (2024), PBK models for 161 orally exposed compounds (mostly pharmaceuticals) were parameterized using a variety of in silico and in vitro based input methods, resulting in 78% of median C_max_ predictions being within fivefold of in vivo validation data. In two separate studies, 44 compounds were assessed using a generic model for rats and humans with different combinations of NAM-based input parameters. Median C_max_ predictions after a single oral exposure were within a factor of 5 for 77% and 50% of the compounds for humans and rats, respectively (Punt et al. 2022a, 2022b). These studies address the sensitivity of a generic PBK model workflow predictivity towards the type and quality of input parameters.

This study builds on the aforementioned studies and assesses the applicability of a generic PBK modeling workflow across chemicals with diverse kinetic properties. A literature review of PBK models for single dose oral exposure gave insight into their goodness-of-fit, as well as most frequently used assumptions including the applied model structures and ADME parameters. Based on the reviewed publications, the most commonly used input parametrization methods were collected and combined into one “generic” PBK workflow.

Estimates from the generic PBK workflow were benchmarked to their respective published models and in vivo ADME validation datasets. This analysis revealed the impact of chemical properties on the performance of the generic model workflow and was used to quantify the uncertainty of predicted versus observed kinetic data. A more detailed analysis of chemicals with less accurate predictions indicated the limitations of the generic PBK workflow and is used to propose its integration into a tiered assessment framework.

## Material and methods

### Study selection

The PBK model database of Thompson et al. (2021) was screened for generic PBK models, which applied single oral exposure, well-defined exposure scenarios and provided high-quality reference in vivo ADME studies on humans or rats. Physiologically based kinetic (PBK) models were classified by their parametrization strategy. Generic PBK models are parameterized exclusively using in vitro ADME parameters, while hybrid PBK models combine compound specific in vitro ADME parameters with parameters fitted to in vivo data (i.e., calibrated against in vivo observations). Our evaluation of 204 publications yielded 52 useful publications for this study, including 58 PBK modeling workflows for 51 chemicals (Annex 1).

For each of these identified studies, our review workflow systematically collected model assumptions, input parameters and evaluated goodness-of-fit metrics. The workflow extracted key summary pharmacokinetic parameters like C_max_, T_max_, and AUC (when available), alongside the corresponding plasma concentration–time curves. A relational database captured and organized all records to support subsequent analyses. In instances where plasma concentration–time curves were not directly reported in the literature, data were extracted from graphical representations using the web-based tool WebPlotDigitizer (Rohatgi). Additionally, we employed the integral function "*sintegral*" from the R package Bolstad2 to address gaps in AUC data and ensure comprehensive data representation.

### Generic PBK model workflow

**Fig. 1:**
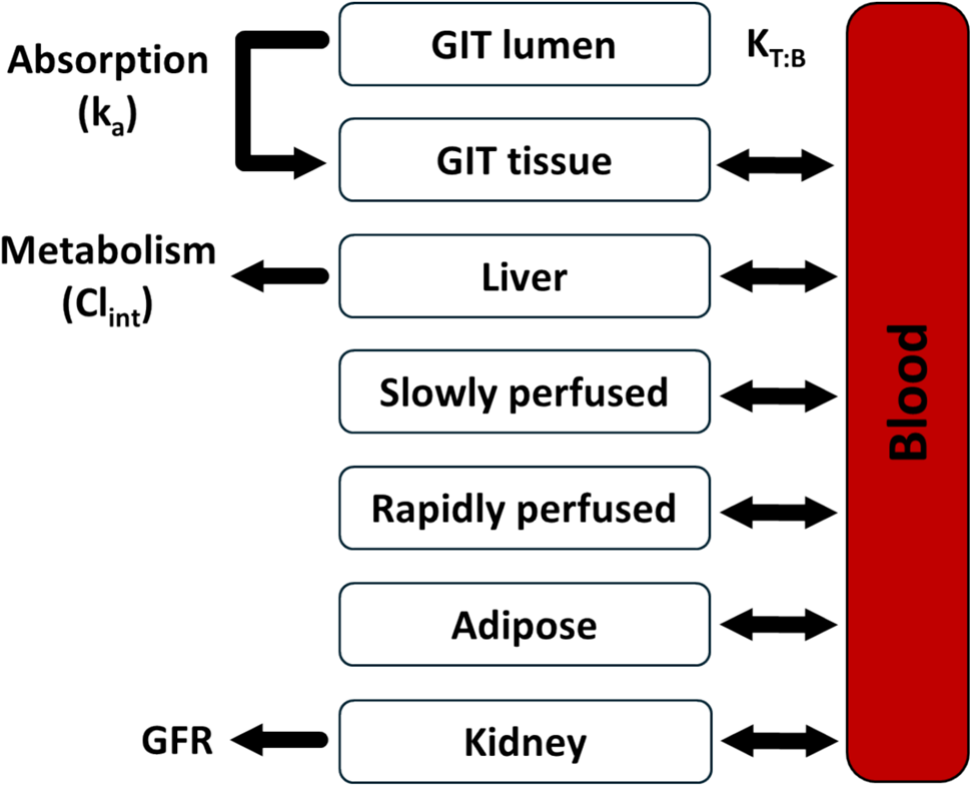
Generic oral PBK model structure Rat and human organisms are subdivided into eight compartments, corresponding to the main tissues and organs in the body. K_a_ refers to the absorption rate constant, Cl_int_ to the intrinsic hepatic clearance, K_T:B_ to the tissue-blood partitioning and GFR to the glomerular filtration rate of the kidney.

The generic PBK model structure combines the most frequently used assumptions and workflows of bottom-up models and is in line with generic models and platforms such as the *httk* R package (Pearce et al. 2017) and *TKPlate* (Dorne et al. 2023). It consists of eight compartments, representing the main tissues and organs integral to rat or human physiology (Fig. 1). Gut lumen is coded as the exposure route in the model, while liver, kidney, and adipose tissue are modeled separately for their clearance mechanisms (intrinsic hepatic clearance (Cl_int_), glomerular filtration rate (GFR)) or a significant difference in cellular tissue composition (adipose tissue), respectively. All other organs and tissues are lumped together on the basis of their relative physiological perfusion (*slow perfusion* < *0.5 ml/min/ml tissue* > *rapid perfusion*).

Data on the underlying physiology of the human or rat subject in the model is based on published species data by the ICRP (2002) and Brown et al. (1997), respectively. For humans, a 73 kg male and for the rat, a 350-g male represent the majority of the studies and are therefore assumed for all model workflows. The PBK model is implemented as an R script and its differential equations are solved with the “deSolve” R package (Soetaert et al. 2010). As input parameters, the model requires basic physicochemical (PhysChem) parameters as well as three compound-specific kinetic indicators, i.e., fraction unbound to plasma (f_up_), apparent permeability (P_app_) and Cl_int_ derived from primary hepatocytes or microsomes (Table 1). During this study, in vitro data was prioritized over in silico predictions, even when the data addressed different species.

**Table 1:** Input parameters for the PBK model workflow

Input parameter	Source	Method	No. of chemicals
*Physchem properties*			
logP_ow_, pKa, H-bond donor/acceptor	ACD labs (GALAS)	In silico	26
*Intestinal uptake*			
Caco-2 P_app_	Literature research	In vitro	18
Caco-2 P_app_	OPERA predictor	In silico	8
*Partition coefficient*			
Rodgers and Rowland	Rodgers and Rowland (2006)	In silico	26
*Hepatic (intrinsic) clearance*			
Derived from primary hepatocytes	Derived from the CompTox database, OPERA predictor database and literature research	In vitro	19
Derived from microsomes	Literature research	In vitro	5
Derived from primary hepatocytes	ADMET predictor	In silico	2
*Fraction unbound in plasma*			
f_up_	Derived from CompTox database and literature research	In vitro	21
f_up_	ADMET predictor	In silico	5

Percepta ACD labs predictor (ACD/Labs Release 2019.1.1. (Build 3200, 17Jul 2019)) generated PhysChem properties of all chemicals in the dataset.

The PBK model describes absorption from the gastrointestinal tract by first-order kinetics derived from the P_app_ measurements in Caco-2 cells. The P_app_ is extrapolated to the effective permeability (P_eff_) as described by Sun et al. (2002) (Eq. 1).

1 $$\log \left( {P_{effhuman} } \right) = 0.4926 * \log \left( {P_{app} } \right) - 0.1454$$

For rats, P_eff_ can be estimated from human P_eff_ with the correlation provided by Wahajuddin et al. (2011) (Eq. 2).

2 $$\log \left( {P_{effrat} } \right) = \frac{{\log \left( {P_{effhuman} } \right) + 0.1815}}{1.039}$$

Uptake processes are divided into fraction absorbed (F_a_) in the gut and the absorption constant (k_a_). The absorbed fraction in the gut is determined by the method represented in Yim et al. (2020) (Eq. 3).

3 $$Fa = 1 - e\left( {\frac{{ - 2P_{eff} *T_{res} }}{R}} \right)$$

The residual times in the small intestine (T_res_) for humans and rats are 4 (ICRP 2002) and 1.47 (Grandoni et al. 2019) hours, respectively. Small intestinal radius (R) depicts the weighted mean of measurements over the small intestine and is 3.05 (ICRP 2002) and 0.18 (Komiya et al. 1980) cm for humans and rats, respectively.

Absorption from the GIT lumen into tissue is determined by the absorption rate constant (k_a_) (Krishna and Yu 2008) (Eq. 4).

4 $$k_{a} = 2 * \frac{{P_{eff} }}{R} * \frac{3600}{{10000}} \left( {h^{ - 1} } \right)$$

Hepatic intrinsic clearance (Cl_int_) is scaled up to the liver properties of the modeled species using IVIVE. For hepatocyte assays, hepatocellularity (hepatocytes per gram liver) is used for scaling, which has been documented in the literature for humans; 99 × 10^6^ cells per gram liver) and rats (Sohlenius-Sternbeck (2006); 117 × 10^6^ cells/g liver). Microsome assays are scaled by the protein content of liver cells which have been documented earlier for humans (Ye et al. 2016) and rats (Houston and Galetin 2008) at 40 and 45 mg protein/g liver, respectively.

5 $$Cl_{H} = \frac{{Q_{liver} \cdot Cl_{int} \cdot f_{ub} }}{{Q_{liver} + Cl_{int} \cdot f_{ub} }}$$

The Clint is then inputted into the well-stirred liver model by Pang and Rowland (1977), assuming a single compartment where compound concentrations are in an instantaneous equilibrium and only unbound fractions are available for metabolism (Eq. 5).

A default value was used for the blood-to-plasma ratio, which is assumed to be 1. Renal clearance via glomerular filtration rate is set to 125 ml/min for humans (ICRP 2002) and scaled to the respective bodyweight for rats using values provided by Carrara et al. (2016) (Eq. 6).

6 $$GFR_{rat} = BW_{rat} *9.9*\frac{60}{{1000}} \left( \frac{L}{h} \right)$$

The *flow-limited* hypothesis is frequently used in generic PBK models, which assumes that the concentration gradient between plasma and tissue and the resulting mass exchange is limited only by blood flow. This assumption is based on the premise that mass transported by the blood is rapidly exchanged with perfused tissues, and that the rate-limiting step of this process is the flow rate rather than diffusion. Within our generic model, this hypothesis is integrated by using the partition coefficient and the difference between blood concentrations flowing to the tissue (${C}_{art}$) and coming from it (${C}_{ven}$). The concentration in tissues (${C}_{i}$) is assumed to be in constant equilibrium with the unbound plasma concentration, determined by the respective tissue partitioning coefficient (${K}_{t2p}$) and blood-to-plasma ratio (B:P) (Eq. 7).

7 $$Cven_{i} = \frac{{C_{i} }}{{K_{{t2p_{i} }} }}*B:P_{Ratio}$$

### Parametrization and characterization of the generic PBK model.

Experimental data were sourced from high-quality databases like CompTox (v2.4.1; Williams et al. (2017), https://comptox.epa.gov/dashboard/) and OPERA (v2.9.1; Mansouri et al. (2018)) and peer-reviewed publications (Table 1). In the absence of experimental data, the ADMET (v12.0.0.6; ADMET Predictor®) or OPERA software filled data gaps. Information about the dosage and dosage type (absolute or relative to body weight) is sourced from the in vivo data as input parameters.

The generic PBK model was written in R (v4.4.1; R Core Team (2024)), solved with the solver functions of the deSolve package (v1.4; Soetaert et al. (2010)) and visualized with the plotly package (v4.11.0; Sievert (2020)) (S1).

Running the generic PBK model workflow resulted in time resolved concentrations of all compartments and plasma C_max_, T_max_ and AUC. By comparing these metrics to validation data, the quality of model predictions is analyzed. Goodness of fit is analyzed by the factors of three and within three to ten, indicating a good fit and approach, respectively.

A Morris sensitivity analysis workflow quantifies the impact of each uncertain parameter on model performance and outputs (Morris 1991). Two indices are used to rank input parameters for each study: µ*, which is the arithmetic mean of absolute values indicating overall impact; and σ, which is the standard deviation depicting linearity and interactions with other parameters. The endpoint chosen to address sensitivity towards is plasma concentration as an indicator of bioavailability and the most sensitive parameters for each workflow are analyzed.

## Results

### Literature review on NAM data used in bottom-up PBK models

From the Thompson et al. (2021) database and targeted literature searches, 204 publications were gathered which describe generic or hybrid PBK models for humans or rats, with oral route of exposure. The majority of these published approaches modeled pharmaceuticals (N = 144), frequently focusing on drug-drug interaction (DDI, N = 39), while 58 of these studies characterized the kinetics and bioavailable fraction of 51 chemicals. Overall, only a few of the 204 published studies applied one of the open-source PBK models, httk or PKSIM, 9 in the drug dataset, while none in the chemicals dataset. Most of these studies either developed their own PBK models (N = 94) or used commercial software like GastroPlus (N = 17) or Simcyp (N = 82).

Of the 58 generic approaches addressing chemicals, the greater portion (N = 38) modeled a male human subject, 13 studies also included rats, while an additional 20 studies modeled only rats. Most models (N = 45) used a simple, generic PBK modeling approach that divides the organism into few compartments, i.e., the GIT for uptake, liver and kidneys for metabolism and excretion, adipose tissue for potential accumulation, and subdivides the remaining tissues of the body into rapidly and slowly perfused compartments. Uptake is determined by the fraction absorbed and the absorption rate constant. Hepatic metabolism and GFR-mediated renal excretion are implemented in these models to describe the substance’s elimination kinetics.

Thirteen PBK models incorporated complex mechanisms, such as cytochrome P450 (CYP) metabolism with Michaelis–Menten kinetics, or refined compartmentalization of organs (e.g., liver or intestinal tract). Here, complexity is defined as extending beyond the basic model structure to include substance-specific mechanisms.

**Fig. 2:**
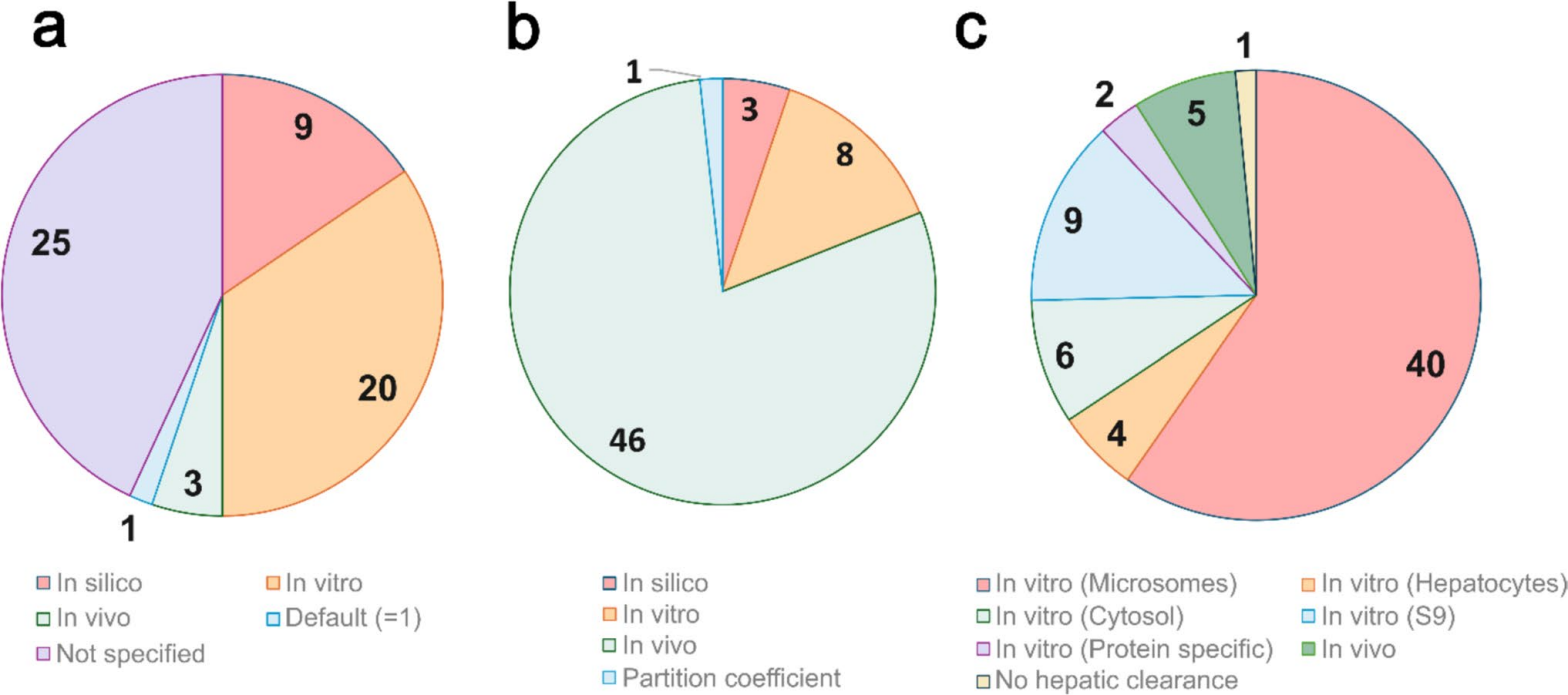
Overview of generic PBK models input parametrization Fraction unbound in plasma is defined close to half of all models (**A**). Absorption kinetics are frequently fitted to in vivo kinetic data, with only a limited number of model approaches utilizing cell assays for prediction (**B**). Hepatic clearance demonstrates a multifaceted utilization of in vitro assays, with microsomes being the most predominate (**C**).

The dataset also included hybrid PBK models which were found in both groups of simple and complex models. Of the 58 PBK models, 50 included at least one in vivo derived ADME parameter, whereas merely five relied solely on a limited set of chemical-specific in vitro ADME inputs (e.g., fraction unbound, hepatic clearance, intestinal barrier permeability). The distribution of ADME parameters is detailed below (Fig. 2).

Fraction unbound in plasma was used in 33 models (57%, Fig. 2A); most often derived from in vitro measurements (N = 20; 34%) or in silico algorithms (N = 9), while few models used in vivo data (N = 3) or did not specify (N = 1).

Absorption is the most prominent in vivo driven parameter with 46 models (79%) using rat or human plasma data to fit absorption (Fig. 2B). Eight (14%) and three (5%) PBK workflows integrated in vitro and in silico data, respectively. The predominant model for in vitro workflows is the Caco-2 barrier model, for whose endpoint in silico models also exist.

The number of liver models exceeds the study count, as some workflows integrated multiple hepatic models to assess hepatic clearance. Hepatic clearance is predominantly parameterized using in vitro methods while only five models integrate in vivo derived metabolism (Fig. 2C). Several in vitro and in silico methods can be used to measure intrinsic hepatic clearance, and this fact is reflected in our analysis. Most models (40; 69%) integrate human liver microsomes, followed by S9 (9; 16%) and liver cytosol (6; 10%). The in vitro method selection for kinetic modeling was tailored to the research question: liver microsomes/S9 were used for phase I, and PHH/liver cytosol/S9 for phase II metabolism.

In our dataset, diverse assumptions and input strategies are used across the generic PBK models to best represent compound-specific ADME properties, with NAM data frequently combined with in vivo fitted parameters. Owing to this diversity, a consistent set of sensitive in vitro/in silico parameters could not be identified, nor could their impact on the performance of a generic PBK model.

### Parametrization of a generic model workflow

Reliable in vivo validation data were available for 49 studies assessing 26 chemicals.

Most compounds had experimental data on the intrinsic hepatic clearance, taken from the CompTox and OPERA databases or published papers (Supplement Table). In silico predicted values per species were used for aristolochic acid and dibromoacetic acid. Twenty-two compounds had experimental data on their unbound fraction in human plasma. Missing data for four compounds were predicted using ADMET predictor. For intestinal permeability, 21 compounds had in vitro data from a published database or were identified via literature search. Five lacked experimental data, and data gaps were filled by in silico models from the OPERA software.

### Model performance

**Fig. 3:**
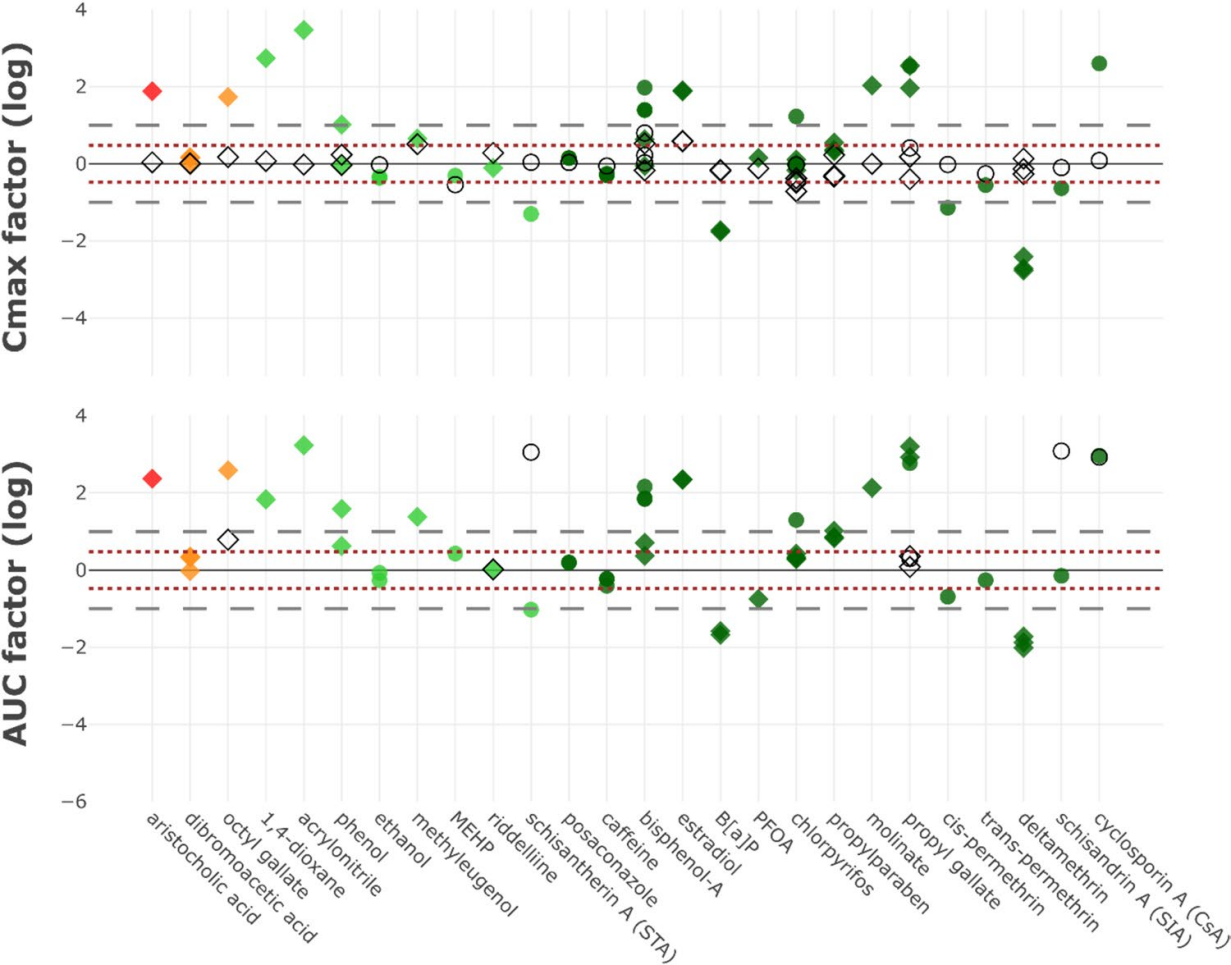
Comparison of C_max_ and AUC of PBK model publications to generic model´s output Compounds are sorted by the certainty of input data, ranging from all parameters predicted (red), 1 (orange), 2 (light green) and all (green) in vitro input parameters measured. Factors for C_max_ and AUC of published models (black open symbols) to the respective in vivo studies are compared to generic models’ outcomes for rat (diamond) and human (circle). Indicators of goodness of fit are depicted by dotted (factor 3) and dashed (factor 10) lines. Approximately half of model runs of chemicals are predicted well within a factor of 10, other predictions surpass a factor > 10. Compounds within that range are generally more likely to be overpredicted.

Performance of the generic PBK model was assessed by comparing the model-predicted to the in vivo measured ADME parameters i.e., C_max_, T_max_ and AUC. Next, the predicted data from the generic model workflow were compared to those from the published PBK models, which often used fitted input data or more complex PBK modeling workflows (Fig. 3).

For the 26 compounds, exposure scenarios of 49 studies were replicated in the generic modeling workflow (Table 2). The results for each chemical are averaged over the number of studies, and an overview of the predictive power of the models is presented in Table 2.

**Table 2:** Overview of model’s performance on each chemical for C_max_, T_max_ and AUC Results are presented as the total average of all studies for their respective chemical.

Factor to in vivo study	< 3	3—10	> 10
C_max_	8 (31%)	5 (19%)	13 (50%)
T_max_	12 (46%)	8 (31%)	6 (23%)
AUC	8 (31%)	5 (19%)	13 (50%)

The C_max_ of 8 out of the 26 compounds was predicted within a factor of three, while five predictions ranged between three- and ten-fold. The remaining 13 compounds were predicted with a factor higher than 10, most of which were overpredicted (69%, Fig. 3). T_max_ predictions showed a good overall predictivity with almost half of all model estimations being within a factor of three (S2).

All published PBK models predict a C_max_ within a factor of 10, most of them within a factor of three (Fig. 3, black open symbols). The largest differences between the generic and the published model are observed for deltamethrin and acrylonitrile, under- and overpredicting C_max_ by factors of about 600 and 3000, respectively. The next section analyses the differences between both approaches.

For most chemicals in our dataset, C_max_ and AUC predictions follow a similar trend (Fig. 3). Of the 26 compounds, the PBK model predicted eight (31%) within a factor of three, five (19%) within a factor of three to ten and 13 (50%) above a factor of ten for both metrics. Data on AUC predictions were rarely (five compounds) provided in published PBK models. While the published models performed well in terms of C_max_ predictability, the few AUC values depicted showed greater deviation, which was more in line with the performance of a generic model. Another observation was the potential efforts of authors to validate and optimize the published model for C_max_ but not for other metrics such as AUC.

### Chemical analysis

Regression analysis was performed to identify trends based on physicochemical properties including logP_ow_ and ionization and in vitro measurements of f_up_, P_app_ and Cl_int_. The analysis aims to identify possible correlations and evaluate the applicability domain of generic PBK modeling. Model outputs that were above a factor of 10 were further analyzed for refinements of these published models relative to a generic workflow.

**Fig. 4:**
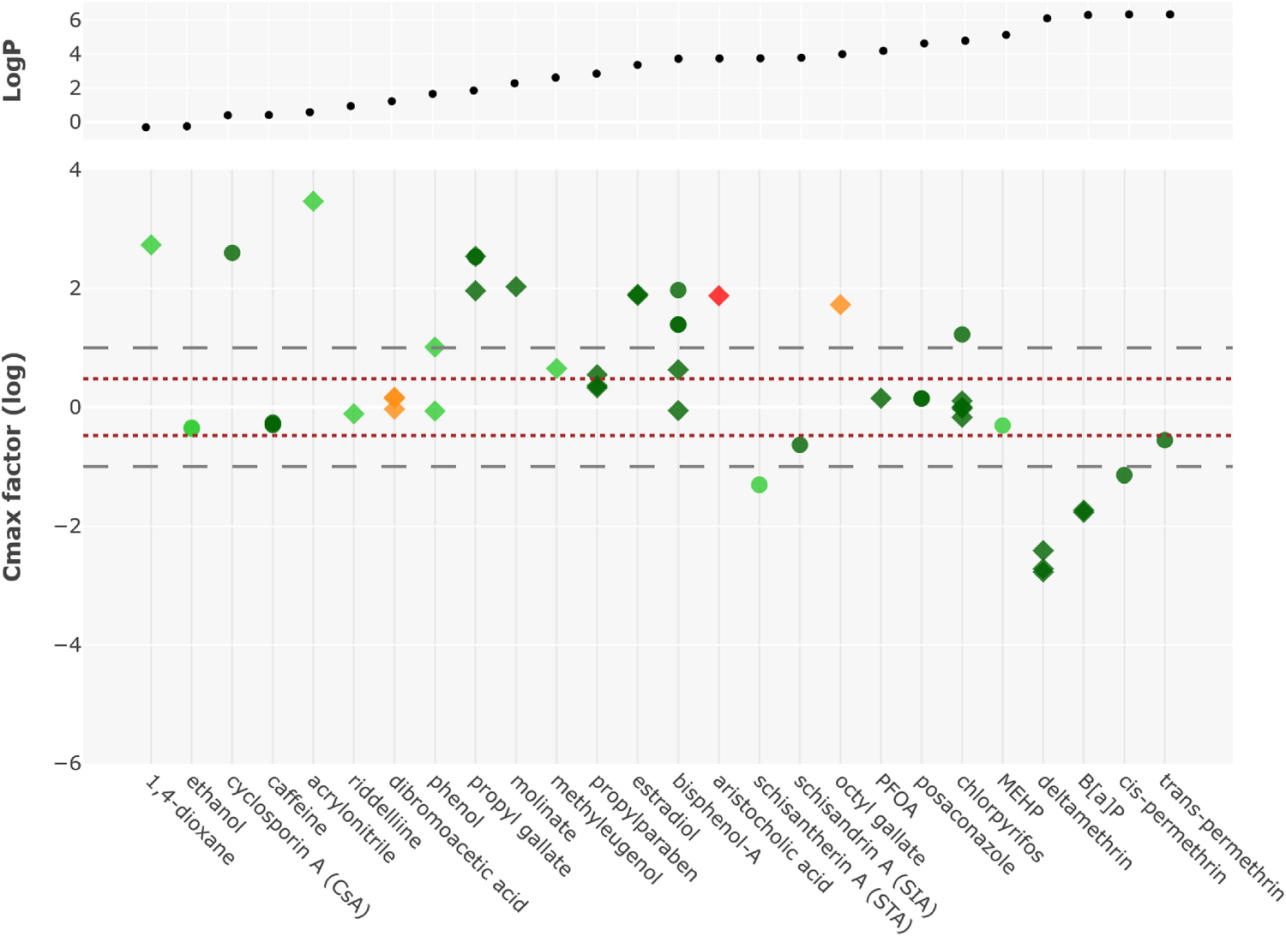
Analysis of model’s performance on C_max_ with regards to logP_ow_ value of chemical Values range from −0.3 to 6.33 and show a trend in models C_max_ estimations. An increase of logP_ow_, indicating higher lipophilicity, results in the generic model initially overpredicting, subsequently underpredicts the maximum concentration in plasma. This effect is seen starting at logP_ow_ values above 4.

The octanol–water coefficient logP_ow_ indicates the lipophilicity of a compound as a measure of partitioning between water and octanol phase. Within the modeled dataset of 21 chemicals, this value covers about six orders of magnitude, ranging from -0.3 (1,4-dioxane) to 6.33 (trans-permethrin). A trend in the performance of the PBK model, indicated by the logarithmic factor of C_max_ prediction can be seen when chemicals are ordered by their logP_ow_ values (Fig. 4). While hydrophilic compounds tend to result in an overestimation of C_max_ using the generic PBK model, an increase of logP_ow_ shifts the model’s predictions towards underprediction (logP_ow_ > 4).

Other chemical or in vitro measured TK properties did not show an impact on the predictivity of C_max_, T_max_ or AUC values (S3—S6).

### Differences in generic to published PBK models

Despite exhibiting a good fit in published models, 23 individual model runs for 15 compounds showed a higher than tenfold deviation in C_max_. Most of these deviations are overpredictions (16; 69%) compared to in vivo measured data and are thus conservative (Table 3). The sources of their input parameters are listed below and range from fitting to in vivo data, to in vitro and in silico methods. Further refinement of the published model’s complexity is depicted below.

**Table 3:** Analysis of generic model runs above the maximum plasma concentration factor of ten Model runs are ranked by log10 of C_max_ factor, negative values indicating an underprediction and positive values signify an overprediction. Model refinements deviating from the generic model are listed under refinement. MM = Michaelis-Menten; GIT = gastrointestinal tract, Cl_int_ = hepatic intrinsic clearance.

Substance	Species	C_max_ factor (log10)	Absorption	Plasma binding	Hepatic clearance	Refinement
Deltamethrin	Rat	−2.77	In vivo	In vitro	In vivo	Cytosol and plasma clearance; diffusion-limitation adipose tissue
Deltamethrin	Rat	−2.72	In vivo	In vitro	In vivo	Cytosol and plasma clearance; diffusion-limitation adipose tissue
Deltamethrin	Rat	−2.41	In vivo	In vitro	In vitro	Cytosol and plasma clearance; diffusion-limitation adipose tissue
B[a]P	Rat	−1.77	In vivo	In vivo	In vivo	MM kinetics; diffusion-limitation fat tissue
B[a]P	Rat	−1.73	In vivo	In vivo	In vivo	MM kinetics; diffusion-limitation fat tissue
Schisantherin A (STA)	Human	−1.30	In vitro	In vitro	In vitro	–
cis-Permethrin	Human	−1.14	In vivo	In vitro	In vivo	–
Phenol	Rat	1.01	In vivo	In vitro	In vitro	MM kinetics; GIT, kidney, & lung clearance, S9; cytosol
Chlorpyrifos	Human	1.22	In vivo	In vitro	In vitro	CYP liver metabolism
Bisphenol-A	Human	1.39	In vitro	In silico	In vitro	Unbound fraction Cl_int_
Bisphenol-A	Human	1.39	In vivo	In vitro	In vitro	MM kinetics; Adapted renal clearance
Octyl gallate	Rat	1.73	In silico	In silico	In vitro	Unbound fraction Cl_int_; S9 liver
Aristolochic acid	Rat	1.88	In vivo	Not specified	In vitro	–
Estradiol	Rat	1.88	In vitro	In vitro	In vitro	S9 liver
Estradiol	Rat	1.90	In vitro	In vitro	In vitro	S9 liver
Propyl gallate	Rat	1.96	In silico	In silico	In vitro	Unbound fraction Cl_int_; S9 liver
Bisphenol-A	Human	1.97	In vitro	In silico	In vitro	Unbound fraction Cl_int_
Molinate	Rat	2.03	In vitro	In vitro	In vitro	Unbound fraction Cl_int_; adapted renal clearance
Propyl gallate	Human	2.53	In vivo	In silico	In vitro	Unbound fraction Cl_int_; S9 liver
Propyl gallate	Rat	2.54	In vivo	In silico	In vitro	Unbound fraction Cl_int_; S9 liver
Cyclosporin A (CsA)	Human	2.60	In vitro	In vitro	In vitro	CYP liver metabolism; adapted renal clearance
1,4-dioxane	Rat	2.73	In vivo	In silico	In vitro	Adapted renal clearance
Acrylonitrile	Rat	3.47	In vivo	In silico	In vitro	Adapted renal clearance

The majority (52%) of those published PBK workflows use in vivo derived absorption data, while plasma binding and hepatic clearance are often integrated by in vitro models. Fitting absorption to in vivo data seems to explain much of the differences in the improved fit of published models to study data, as most other assumptions are shared with the generic workflow. Further improvements if identified come from refinements in hepatic clearance and other model assumptions.

For compounds such as phenol and propyl gallate, which undergo phase 2 metabolism, the model workflows integrated hepatic S9 and cytosol model measurements. The metabolism of esters, as in the case of deltamethrin, is integrated by the kinetics of the plasma stability assay. Additionally, renal clearance adapted from in vivo urine data refined the model approach of 1,4-dioxane, molinate, acrylonitrile, cyclosporin A and bisphenol-A.

Deltamethrin and B[a]P are examples of highly lipophilic compounds with logP_ow_ values of 6.1 and 6.23, respectively. Here, an accumulation of the chemical in the adipose tissue is a known mechanism and integrated by the PBK model with a high partitioning coefficient. In their published PBK models, a refined assumption is the diffusion-limitation of substance to the adipose tissue (Foth et al. 1988; Song et al. 2019). Due to its high lipophilicity, a flow-limited partitioning assumptioninstantaneously exchanges mass in a higher rate than diffusion potentially allows, resulting in a lower bioavailability in the early phase.

Some model workflows for molinate, propyl and octyl gallate as well as bisphenol-A also adapt the free fraction of the in vitro hepatic clearance assay for the free fraction inside the system.

Sensitivity and uncertainty analysis of the generic PBK model.

The impact of different input parameters with regard to their uncertainty and sensitivity towards model predictions indicates the overall uncertainty and individual contributions, respectively. P_app_, f_up_ and Cl_int_ values were extracted from one source each, not all of which indicated a standard deviation. To assess uncertainty for all chemicals in a comparable manner, an artificial 0.5–twofold uncertainty range, as recommended in the OECD (2021) guidance when the uncertainty remains unknown, was applied via one-at-a-time (OAT) input variation, with the exposure scenario and species data held constant. The predicted tissue partitioning parameters were similarly varied to reflect uncertainty in the in silico method.

**Fig. 5:**
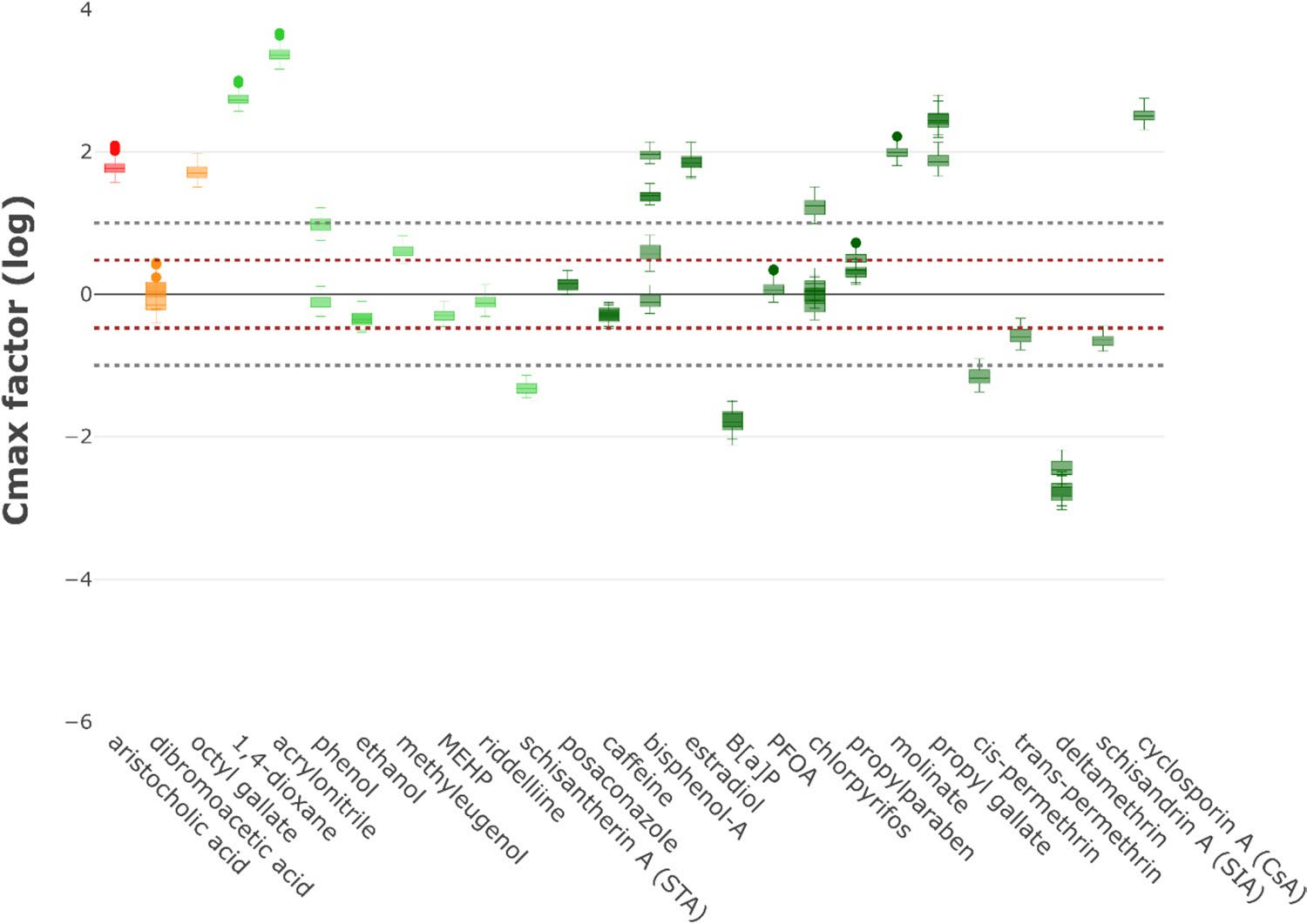
Distribution of C_max_ values for uncertainty analysis An artificial 0.5 to twofold range is assumed for all input parameters including LogP_ow_, fup, P_app_, Cl_int_ and partitioning coefficients for all tissues. The boxplots indicate the variety of C_max_ estimations per each chemical and model. Although all parameters varied within the same factors, results vary compound and study specific.

The modeled uncertainty of all studies resulted in 10,290 C_max_ values (210 runs for each of the 49 studies) with their distribution depicted in boxplots (Fig. 5). Ranges of values are substance- and exposure scenario specific andwide ranges of C_max_ values are observed for dibromoacetic acid and propyl gallate.

A Morris sensitivity analysis identified five unique input parameters as consistently exhibiting the highest sensitivity across all 26 chemicals and their PBK studies. The most frequently sensitive parameters were fup (N = 9; 35%), P_app_ (N = 4; 15%) and partitioning into adipose (N = 6; 23%), intestinal (N = 5; 19%) and muscle (N = 2; 8%) tissues (S6). Depending on the exposure scenario and the species exposed, the most sensitive parameter for the same chemical was always the same, while the second and third most sensitive parameters sometimes varied (see S6). This can be seen in the example of phenol and propylparaben exposure in rats, where the most sensitive parameter is shared by the workflows, but different dosing seems to influence the second and third most sensitive parameters.

For the same chemical, depending on exposure scenario and species exposed, the most sensitive parameter was always the same, while the second and third most sensitive parameters in some cases varied (S7).

## Discussion

Generic PBK models are essential for NGRA to derive human equivalent doses within the process of IVIVE. Accordingly, their chemical domain of applicability and associated uncertainties require thorough characterization. Our review of widely used generic PBK models revealed substantial heterogeneity in model structures, assumptions and parametrization, with little consensus across approaches. A few assumptions seem to be state-of-the-art, e.g., the scaling procedure of in vitro hepatic clearance from protein content (microsomes) or the number of hepatocytes to the in vivo liver clearance. These models are known to have their advantages and disadvantages with respect to handling and in vivo resemblance (Ooka et al. 2020).

Human microsomes are often derived from larger donor pools and are therefore less variable compared to, e.g., primary human hepatocytes (Bale et al. 2016). The choice of the appropriate in vitro model is often driven by scientific questions, for instance whether phase I and phase II metabolism need to be captured. Most published models did not aim for an animal-free paradigm; rather, they integrated all available evidence, frequently incorporating in vivo ADME data to optimize the parameter estimates of the bioavailable compound fraction. This reliance likely explains the large number of PBK models for chemicals that include in vivo ADME data.

Overall, our analysis confirms that generic PBK modeling incorporating only a few measured in vitro ADME parameters and generic kinetic mechanisms possesses the capacity to predict the kinetics of chemicals with a relatively high degree of predictivity. For approximately half of the analyzed compounds (48%), the generic model with a fixed input parametrization workflow predicted the Cmax within a tenfold range. Furthermore, 30% of the total chemicals were within a factor of 3, indicating a robust fit for the models’ C_max_ performance. Similar outcomes were observed with regard to the AUC performance. For the T_max_ predictions, the model achieved an even better fit with about 77% of chemicals falling within a tenfold change. The bioavailability of most of the chemical compounds in the studies that are predicted to fall within a factor of ten or higher is overestimated. An overestimation of the bioavailable fraction of a test compound is conservative in the context of safety assessment as an IVIVE would predict a lower corresponding external dose.

Comparable studies on generic PBK models for humans and rats showed similar results with most study outliers being overpredicted and none underpredicted by more than a factor of 5 or 10, respectively (Punt et al. 2022a, 2022b). In their analysis, 44 compounds – 37 drugs and seven chemicals in the human study, and 13 drugs and 31 chemicals (mainly insecticides and herbicides) in the rat study — were assessed using a generic PBK workflow. This resulted in 22 (50%; rat) and 34 (77%; human) compounds being predicted to be within a factor of 5, respectively. However, while our study focuses on implementing a minimal input workflow with commonly used input parameters, Punt et al. varied the input parameters using multiple models and, if accessible, collected several in vivo validation datasets, representing a higher degree of study complexity by the inclusion of this variability.

Published models of most chemicals showed a better predictability than the generic simple modeling workflow. This is no surprise, as many of the literature models integrate in vivo data or more complex kinetic mechanisms to improve the model outputs for specific compounds. To address this, some chemicals with a relatively large difference in the models’ performance were also further analyzed in our study.

In vivo data are used for various purposes, including model validation and parameter fitting for the absorption rate constant, hepatic metabolism and renal clearance (Table 2). Here, the focus is on the peak concentration C_max_ rather than the total bioavailability as represented by the AUC (Fig. 3). By fitting data and input parameters, mechanisms and assumptions that are not integrated into the generic model but that are relevant for the depiction of the compound’s kinetics are compensated. For chemicals such as 1,4-dioxane and acrylonitrile, for example, integrating in vivo data to fit and refine model parameters significantly improved the fit. However, this approach does little to advance animal-free NAM-based PBK modelling and extrapolation to different exposure scenarios.

Generic models, which are built on in vitro and in silico parameters, refine their workflow by integrating more detailed and more complex inputs and model assumptions that cover the relevant ADME properties of their respective chemicals.

A variety of hepatic clearance models are available, ranging from relatively inexpensive, high-throughput models to complex, expensive ones (Ooka et al. 2020). For example, the published PBK model for propyl gallate incorporates an S9 liver mix to simulate hepatic clearance (Punt et al. 2021). In comparison to the generic model workflow of our study, they observed an improved fit of Cmax and AUC which could be the result of a more suitable hepatic model, as propyl gallate is known to undergo phase II metabolism which is better captured by S9 liver models than by the microsomes used in this study (Daniel 1986).

The choice of suitable models representing in vivo situations and parametrization methods is crucial for generic PBK models. In our study, model run parametrization included three key TK parameters, in line with the most common approaches used in generic models found in the literature and resulted in reasonable validation for half of the modeling workflows. Model performance or improved goodness of fit of model runs due to different parametrization methods was outside the scope of this study but has been documented in earlier studies (Geci et al. 2024; Punt et al. 2022a).

The influence and sensitivity of parametrization for generic PBK modeling for human oral exposure was recently analyzed by Punt et al. (2022a, b). As crucial criteria for ensuring the model’s suitability based on in vivo validation data of 44 compounds, they identified in vitro measurements of hepatic clearance in hepatocytes or S9 fractions, the partitioning algorithm of Rodgers and Rowland (2006), and the prediction of the fraction unbound in plasma by Lobell and Sivarajah (2003).

Geci et al. (2024) parameterized a high-throughput, generic PBK model using various in silico, in vitro, and in vivo algorithms and parameters for intravenous and oral exposure of 143 and 169 compounds, respectively. These compounds were primarily drugs. The main outcome was that C_max_ was within a factor of ten in 87% of all modeled compounds. Inputs for logP_ow_ and Cl_int_ had the greatest impact on model performance, and prediction of highly lipophilic compounds appears to be most accurate in workflows incorporating the partitioning models of Poulin and Theil (2002) and Berezhkovskiy (2004). This may partly explain why compounds with logP_ow_ values greater than four were underpredicted in this study. In silico predictions are used to fill gaps in hepatic clearance data. To date, the publicly available data on in silico models for hepatic clearance show a rather poor correlation to data measured in vitro, so that their reliability, and consequently their usefulness for PBK models, seems to be limited (Pradeep et al. 2020).

Both aforementioned studies by Geci et al. (2024) and Punt et al. (2022a) share the idea of assessing generic and high-throughput modeling towards their predictability and best approach. Their results are in line with ours, showing the capability of generic modeling for a set of diverse compounds. However, their studies include drugs with generally more robust data. Hence, many algorithms and assumptions are derived from existing in vivo data and are unclear in their applicability for chemicals. Additionally, they compared various combinations of input parameters and demonstrated significant differences in model results. The parametrization of a NAM-based PBK model can draw from a large pool of in silico and in vitro methods. Selecting the most suitable parameters to explain in vivo studies provides proof of concept, but it limits the scope of PBK models to available validation data. Regulatory guidance from the WHO (2010) and OECD (2021) recommends several metrics for chemical risk assessment without the need for in vivo validation data. These include read-across, sensitivity and uncertainty analysis, and the validity of biological and theoretical basis. As this study shows, uncertainty and sensitivity are chemical- and scenario-specific and must be addressed individually. Even when uncertainty is integrated into the input parameters, the results showed that models that under- or overpredict by a factor of more than 10 remain in their predictability class and do not indicate the potential for improvement. This shifts the focus of improvement from parametrization to missing mechanisms and adaptations of model assumptions.

Missing mechanisms in the generic PBK model workflow were identified through chemical analysis and by comparing published data with refined PBK models. Most adaptations of published PBK models that significantly improve prediction focus on input parametrization, such as considering the most suitable hepatic model, adapting renal clearance, and addressing difficult compounds, such as highly lipophilic ones, with refined model assumptions and in vitro assay corrections (Table 3). The remaining mechanisms that contribute to a chemical’s kinetics but are not considered in the published model are identified and discussed below.

One example of such mechanistic basis is enterohepatic circulation, leading to a higher extent of bioavailability, as hepatic conjugation and intestinal deconjugation prolongs a chemical’s systemic exposure and elimination half-life (Malik et al. 2016). A recent study by Alpizar et al. (2024) analyzed the importance and impact of EHC on models’ AUC performance. Chemicals that undergo EHC show an increase in permanence by a higher t_1/2_ elimination half-life, resulting in an increase of predicted AUC. Compounds in the current study with a high underprediction (factor > 10) of AUC are B[a]P and deltamethrin. Benzo[a]pyrene is known for its enterohepatic circulation whereas for deltamethrin no studies indicating this mechanism have been found. Additionally, propyl gallate is metabolized by gut microbiota which could affect the model’s result (Javaheri-Ghezeldizaj et al. 2023). Another compound undergoing metabolism by gut microbiota is daidzein (Soukup et al. 2021), whose C_max_ is overpredicted in a previous study by a factor of 15.7 (taken from supplementary data; Punt et al. (2022b)).

The bioavailability of a compound is represented by the surrogate parameter, AUC. The extent of a chemical substance’s uptake and distribution within an organism is subject to several variables. These can include accumulation of lipophilic compounds in adipose tissue and enterohepatic circulation (EHC) from the liver to the small intestine, resulting in a secondary reuptake of compound. Neither mechanism is integrated in the generic model workflow, although accumulation in adipose tissue is partially accounted for by the partitioning algorithm.

High lipophilicity is also known to potentially cause plastic binding, resulting in an overestimation of the free medium concentration in in vitro assays (Proenca et al. 2021). For this reason, in vitro kinetic models such as VIVD (Fisher et al. (2019)) are used to account for the loss of compounds via plastic binding or evaporation. Compounds with high logP_ow_ values are, however, outside their applicability domain, indicating another need for further research.

Relevant kinetic mechanisms and assumptions are difficult to assess without predictive indicators and quantifying measurements. While high lipophilicity may indicate the need for a refined partitioning, the relevance of other mechanisms like pre-systemic metabolism by enterocytes and/or microbiota or active transport and their extent could be more difficult to predict. The ADME kinetics of a chemical are a complex interplay of multiple factors and difficult to address by single measurements. Although chemical classes within this research show some clustering and trend in generic modeling, further efforts should be undertaken regarding predictive indicators and their quantification. As possibly important kinetics already integrated for some chemicals in published PBK models, enterohepatic circulation and gut metabolism are identified. It should also be noted that this dataset only analyses single exposure scenarios. Further research should address the potential accumulation of compounds (like PFOA) resulting from multiple or chronic exposure.

To provide a broader context, it is important to note that this study only assesses a small fraction of the total number of chemicals currently on the EU market. There are approximately 100,000 chemicals currently available, 70.000 of them hardly have any available information on potential environmental or human hazards (Agency 2019). The lack of in vivo kinetics data for the purposes of validation and kinetic characterization limited our evaluation to a small subset of compounds, and the chemical domain analysis would undoubtedly benefit from a greater quantity of data for a broader set of structurally diverse chemicals. In the light of the 3R paradigm, it could be advantageous to include ADME kinetic measurements in already required in vivo studies, wherever feasible, in order to facilitate the optimal utilization of animal experimentation. Additionally, efforts to collect and make in vivo concentration-time data accessible, such as the CvT database curated by Sayre et al. (2020) (US EPA) can further help to standardize data reporting and improve validation of PBK models.

A framework that integrates NAM-based approaches for hazard assessment, kinetic modeling, and exposure assessment is currently needed to improve their implementation in regulatory applications (Wambaugh et al. 2025), such as the interactive NGRA workflow, the Alternative Safety Profiling Algorithm (ASPA), from the RiskHunt3r project (Leist 2025). Many models are constructed for specific chemicals and scenarios. Therefore, guidance on the certainty and validity of generic models when no in vivo data are available is highly important for animal-free kinetic assessments. This study supports the idea that one modeling approach cannot sufficiently describe kinetics for the broad chemical domain (Najjar et al. 2022). For NGRA, the ASPA workflow’s modular and tiered approach offers flexibility in adapting the modeling approach to the problem formulation (Leist 2025). The first tier suggests a simple, generic PBK model with a minimum of NAM ADME data, followed by a more complex, mechanistic PBK model where needed.

## Supplementary Information

Below is the link to the electronic supplementary material.

Supplementary file1 (DOCX 443 kb)

## Data Availability

All collected data on input parameters and published validation data are available in this publication’s supplementary.
