# Supplementary material for: Applicability of next-generation risk assessment generic PBK models and their performance within the chemical domain
Source: Arch Toxicol. 2026 May 25;100(9):3893–906. doi: 10.1007/s00204-026-04443-7 (PMC13453979; doi:10.1007/s00204-026-04443-7)
Supplement: Supplementary file 1 [file 204_2026_4443_MOESM1_ESM.docx]

# Appendix

An overview of the basic structure and the interconnection of the model and their differential mass equations is depicted in the following paragraph. Uptake is defined by the absorption rate constant *k_abs,_* subtracted from the lumen and added the gastrointestinal (GIT) tissue. From here on it is distributed to the liver for the first pass metabolism (assuming a well-stirred liver clearance; Pang and Rowland (1977)) before it reaches systemic availability.

$$dmLumen= -k_{abs}*mLumen$$

$$dmGIT=Q_{GIT}*\left( Cart-Cven_{GIT} \right)+ k_{abs}*mLumen$$

$$Metabolism=Cl_{int}\left( well-stirred \right)*Cven_{liver}*f_{up}$$

$$dmLiver=Q_{liver}*\left( Cart-Cven_{liver} \right)+Q_{GIT}*\left( Cven_{git}-Cven_{liver} \right)-Metabolism$$

$$dmFat=Q_{fat}*\left( Cart-Cven_{fat} \right)$$

$$dmKidney=Q_{kidney}*\left( Cart-Cven_{kidney} \right)-\left( GFR*Cven*f_{up} \right)$$

$$dmSlow=Q_{slow}*\left( Cart-Cven_{slow} \right)$$

$$dmRapid=Q_{rapid}*\left( Cart-Cven_{rapid} \right)$$

S1 Differential equations of generic model compartments.

S2 T_max_ comparison of generic and published models to biomonitoring validation data.

S3 Overview of ionization classes (acids, bases and zwitterions) model performance.

S4 Analysis of fraction unbound in plasma to goodness of fit of generic models.

S5 Analysis of the potential impact of hepatic clearance on generic model results for rat and human.

S6 Overview of apparent intestinal permeability on the results of generic modeling.

S7 Ranking of most sensitive parameter for each model workflow.

| Chemical | Species | Dosage | Dosage unit | Sensitivity parameters ranking | | |
| --- | --- | --- | --- | --- | --- | --- |
|  |  |  |  | 1 | 2 | 3 |
| 1,4-dioxane | rat | 500 | mg/kg | fup | P_muscle | Papp |
| acrylonitrile | rat | 30 | mg/kg | fup | P_muscle | Papp |
| aristocholic acid | rat | 25.3 | mg/kg | fup | P_skin | Papp |
| B[a]P | rat | 0.0008 | mg | P_git | P_liver | Papp |
| B[a]P | rat | 0.001 | mg | P_git | P_liver | Papp |
| bisphenol-A | rat | 10 | mg/kg | P_git | P_liver | Papp |
| bisphenol-A | rat | 100 | mg/kg | P_git | P_liver | Papp |
| bisphenol-A | human | 0.03 | mg/kg | P_muscle | P_skin | Papp |
| bisphenol-A | human | 0.1 | mg/kg | P_muscle | P_skin | P_fat |
| bisphenol-A | human | 0.1 | mg/kg | P_muscle | P_skin | P_fat |
| caffeine | human | 5 | mg/kg | fup | P_fat | P_muscle |
| caffeine | human | 5 | mg/kg | fup | P_fat | P_muscle |
| caffeine | human | 300 | mg | fup | P_fat | P_muscle |
| chlorpyrifos | rat | 100 | mg/kg | Papp | P_skin | P_rest |
| chlorpyrifos | rat | 10 | mg/kg | Papp | P_skin | P_rest |
| chlorpyrifos | human | 2 | mg/kg | Papp | P_muscle | P_skin |
| chlorpyrifos | rat | 50 | mg/kg | Papp | P_skin | P_rest |
| chlorpyrifos | rat | 5 | mg/kg | Papp | P_skin | P_rest |
| cis-permethrin | human | 4.35 | mg/kg | P_git | Papp | P_liver |
| cyclosporin A (CsA) | human | 300 | mg | fup | Papp | P_fat |
| deltamethrin | rat | 0.5 | mg/kg | P_git | P_liver | Papp |
| deltamethrin | rat | 0.25 | mg/kg | P_git | P_liver | Papp |
| deltamethrin | rat | 0.1 | mg/kg | P_git | P_liver | Papp |
| dibromoacetic acid | rat | 25 | mg/kg | fup | Papp | P_skin |
| dibromoacetic acid | rat | 50 | mg/kg | fup | Papp | P_skin |
| dibromoacetic acid | rat | 125 | mg/kg | fup | Papp | P_skin |
| estradiol | rat | 0.02 | mg/kg | P_fat | Papp | P_skin |
| estradiol | rat | 0.08 | mg/kg | P_fat | Papp | P_skin |
| ethanol | human | 600 | mg/kg | fup | P_muscle | Papp |
| ethanol | human | 300 | mg/kg | fup | Papp | P_muscle |
| MEHP | human | 48.5 | mg | P_fat | Papp | fup |
| methyleugenol | rat | 150 | mg/kg | P_fat | fup | Papp |
| molinate | rat | 250 | mg/kg | P_fat | Papp | P_skin |
| octyl gallate | rat | 17.5 | mg/kg | Papp | P_skin | P_rest |
| PFOA | rat | 1 | mg/kg | fup | P_skin | Papp |
| phenol | rat | 1.69 | mg/kg | Papp | P_skin | P_rest |
| phenol | rat | 147 | mg/kg | Papp | fup | P_skin |
| posaconazole | human | 40 | mg | Papp | P_muscle | P_git |
| posaconazole | human | 40 | mg | Papp | P_muscle | P_git |
| propyl gallate | human | 14 | mg/kg | P_fat | Papp | fup |
| propyl gallate | rat | 135 | mg/kg | Papp | P_fat | fup |
| propyl gallate | rat | 14 | mg/kg | Papp | P_fat | fup |
| propylparaben | rat | 3 | mg/kg | P_fat | Papp | P_skin |
| propylparaben | rat | 10 | mg/kg | P_fat | P_skin | Papp |
| propylparaben | rat | 100 | mg/kg | P_fat | P_skin | P_rest |
| riddelliine | rat | 10 | mg/kg | fup | Papp | P_muscle |
| schisandrin A (SIA) | human | 7.2 | mg | P_muscle | P_skin | Papp |
| schisantherin A (STA) | human | 7.3 | mg | P_muscle | P_skin | Papp |
| trans-permethrin | human | 5.65 | mg/kg | P_git | P_liver | Papp |
